# How Does the Amount of a Physical Education Intervention Affect Gross Motor Coordination in Early Childhood?

**DOI:** 10.3390/jfmk7040096

**Published:** 2022-10-28

**Authors:** Giovanni Angelo Navarra, Antonino Scardina, Ewan Thomas, Giuseppe Battaglia, Massimiliano Agnese, Patrizia Proia, Antonio Palma, Marianna Bellafiore

**Affiliations:** Sport and Exercise Sciences Research Unit, Department of Psychology, Educational Science and Human Movement, University of Palermo, Via G. Pascoli 6, 90144 Palermo, Italy

**Keywords:** children, performance, motor skills, kindergarten, physical activity, school

## Abstract

Little is known about the dose–response of physical education interventions on motor coordination in preschoolers. Our aim was to investigate whether the development of motor skills changed depending on different amounts of a physical education program (PEP) in children aged 3–5 years. One hundred forty-five children were recruited from kindergartens and randomly divided into a control group (CG, *n* = 28), which did not perform any PEP, and two intervention groups, which performed 4 h/week (I1, *n* = 78) and 10 h/week (I2, *n* = 39) of a PEP for 16 weeks. Each lesson was set in the form of a programmed game in order to produce fun, thus increasing enthusiasm for participation. Before and after the intervention, locomotor and object control skills and the gross motor development quotient were assessed with the Italian version of the gross motor development test. Both intervention groups showed a significant increase in the motor skills compared with the control group. Moreover, the level of performance was significantly higher after 10 h/week compared to 4 h/week. These findings can be useful for standardizing PEPs in preschool settings so that they can be applied by teachers for planning effective programs for developing motor skills in early childhood.

## 1. Introduction

Early childhood is a fundamental step for child development [1] and physical activity (PA) plays an essential role in this process [2]. According to Canadian guidelines, over a 24-h day, preschool children should spend at least 3 h in a variety of physical activities, including at least 1 h at high intensity [3]. However, numerous studies reported that preschool children are sedentary, not reaching the recommended amount of daily PA [4,5,6]. As is known, the PA in preschool is limited and, furthermore, the level of PA among children living in Europe varies considerably between countries [7]. In Italy, according to ISTAT data for the two-year period 2017–2018, children aged 3–5 years who do not practice any sport or PA in their leisure time represent 46.1% [8]. To expand this negative trend, the development of new digital devices has certainly favored the increase in the sedentary rate of this population, which spends more time with smartphones, tablets, and videogames, increasing the so-called “screen time” [9]. The pandemic situation also adversely affected PA levels in children, making it difficult to practice PA regularly [10]. There are many studies that have shown that performing PA in early childhood brings various benefits, from psycho-physical and cognitive development to educational and social aspect [11]. As for motor development, several studies have reported that the practice of PA improves the level of fundamental motor skills (FMS) by increasing motor competences in childhood [12,13]. FMS are the basic movements and include locomotor skills (running, jumping, galloping, jumping, crawling) and object control skills (bouncing, grabbing, throwing, kicking) [14,15]. They are constituent elements that lead to sport-specific motion sequences and enable life-long movement experiences [16]. In addition, it has been fully demonstrated that motor competence in early childhood is a predictive element for the practice of physical activity throughout life. A longitudinal study [17] evaluated the effects of early motor skills in adulthood and found a positive association between the development of FMS at age 6 and time spent in PA at age 26 in leisure time. This demonstrates the importance of effective interventions to enable children to develop FMS early, thus increasing their motor competence and favoring the establishment of a physically active lifestyle. Children do not develop these abilities naturally through maturation processes [18], but they should be learned, practiced, and reinforced. Precisely in early childhood it is essential to teach these motor skills, especially in those age groups defined as “sensitive”, i.e., evolutionary periods, limited in time, in which there is a favorable training against a specific motor quality. The critical time to develop FMS is in early childhood when movement patterns are developed [18] and before school age, when children begin to participate in games and sports that will require them to use these skills.

A systematic review carried out by Engel et al. [19] showed significant improvements in the competence of preschoolers’ FMS following teacher-led interventions executed for at least three sessions per week. Recent evidence has also exhibited the short and long-term beneficial effects of physical education programs (PEP) not only on the development of motor skill, but also on cognitive development [20,21]. Indeed, an increase in FMS positively influences psychosocial aspects and cognitive functions in preschool children [22,23]. 

For all these reasons, in the early years of the child’s life, the practice of structured physical activities is of crucial importance. To confirm this, Logan et al. have found that structured interventions of PA are more efficient compared with free play activities [18]. However, many studies lack a structured and reproducible PA [21] program in preschool settings that includes specific activities, timing, and duration.

Therefore, the presence of a physical education teacher could be useful in kindergartens for the implementation of PEP aimed at developing FMS in early childhood [24]. Indeed, children spend a good part of their day at school, so the latter could certainly serve as a place to promote PA [25]. The childcare setting provides an environment in which interventions to increase PA can be provided. However, few interventions have involved preschool settings [25]. 

In the scientific literature, there are studies that deal with free play activities but not structured physical activities, or that analyze the amount of structured physical activity that could lead to an increase in the gross motor skills in preschoolers. However, despite the findings that free play could increase engagement, results indicated that it is an insufficient intervention. For this, children should be invited to PA [26] and therefore structured interventions should be proposed. 

The figure of the physical education teacher is missing in both Italian kindergarten and primary schools, and this certainly leads to a decrease in the possibility of developing FMS. It would be necessary, therefore, that children also at school could follow programs of physical education, but still today there is little clarity on the adequate amount of PEP that could lead to an increase in the motor skills of children. Therefore, the aim of this study was to investigate the level of motor skills after 4 h/week or 10 h/week of a PEP of 16-week length in preschoolers.

## 2. Materials and Methods

### 2.1. Participants and Study

A school-based randomized trial was conducted to evaluate the effect of a PEP on preschoolers’ gross motor skills. The calculation of the statistic power with the G*Power software (latest ver. 3.1.9.7; Heinrich-Heine-Universität Düsseldorf, Düsseldorf, Germany) considering a power of 80%, an alpha error of 0.05, an effect size of 0.25 and an ANCOVA test suggests involving a minimum sample size of 158 participants. This study recruited 166 children aged 3–5 years from eight different kindergartens of the Municipality of Palermo, but a number of 145 (55% boys) completed the assessment before and after the intervention. They were randomly divided into three different groups: a control group (CG, *n* = 28; 61% boys) and two intervention groups [(I1, *n* = 78; 54% boys) and (I2, *n* = 39; 54% boys)]. The anthropometric characteristics of the participants are described in Table 1. All parents gave their informed consent for the participation of their children in this study. The study was approved by the Ethical Board of the University of Palermo (N. 2/2018) and conformed to criteria for the use of persons in research as defined in the Declaration of Helsinki (Trial Registration: NCT03454061 retrospectively registered 2 March 2018). All children participated voluntarily and could withdraw from the study at any time. 

**Figure 1:**
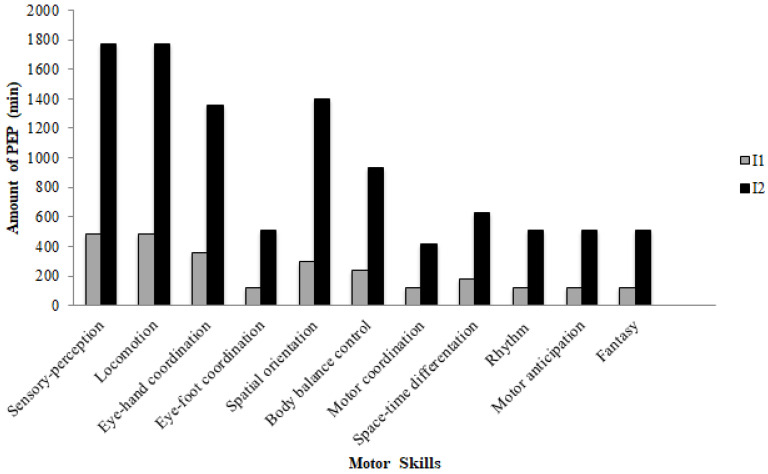
Number of hours spent for the development of the motor skills in I1 and I2 groups.

PEP intervention was carried out in seven kindergartens of Palermo by a team of physical education teachers from February to May of 2019. Physical activity teachers led the PEP and curricular teachers supported the planned activities. Before intervention, they followed a 10-h training course on the objectives, methodology, and evaluation tools of the PEP with the aim of having a homogeneous intervention, allowing a reliable collection of data. This intervention lasted 16 weeks and was carried out for a total of 4 h/week for the I1 group and 10 h/week for the I2 group for a total of 52 and 180 h, respectively [21]. The difference between the I1 and I2 programs was the number of hours spent for the development of the motor skills included in the PEP (Figure 1).

### 2.2. Intervention

PEP was composed of activities aimed at developing body awareness, motor, and perceptive-sensory skills fundamental in preschool children. It was performed by the I1 group in the morning for two days per week, and by the I2 group in the morning and afternoon for five days per week. Each class was structured in three parts: a first phase of body warming up and social interaction (5 min); a central phase (about 50 min); and a phase of cooling down and feedback (about 5 min) in order to relax the children and know their levels of satisfaction. In particular, the motor skills trained in the central phase were: sensory-perceptual and locomotor skills, eye–hand and eye–foot coordination, spatial orientation and rhythm skills. More time was spent for sensory-perceptual and locomotor skills as suggested by the guidelines of the Italian Ministry of Education, University and Research (MIUR) [27]. The detailed description of PEP protocol was reported in our previous study [28]. Each lesson was set in the form of a programmed game in order to produce fun and thus increase enthusiasm for participation. 

### 2.3. Measures

Participants were evaluated for object control and locomotor skills with the Italian version of the gross motor development test (TGMD) [15]. 

This test is divided into two different sub-tests that evaluate different aspects of gross motor development, that is, the control of objects (bounce the ball, catch the ball, catch a ball with a tennis racket, and run while you kick a ball and throw a ball) and the locomotion (which requires subjects to run as fast as possible for 15 m, jump forward, gallop for 10 m, jump on one leg for 5 m, make a long jump, and make small jumps forward and sideways). The scores of the two sub-tests were added and converted into a combined gross motor development quotient (GMDQ).

In order to have a better validity, according to the manual, each child has performed three trials of each skill. For each of these, two different scores were awarded: “1”, when a performance parameter was performed twice in three, or a grade “0”, when a criterion was not respected or was incorrectly played twice in three. The total sum of scores obtained for each individual item (maximum total score 48) was converted into standard scores based on the child’s age level. The gross motor development level based on the GMDQ scores suggested by the manual was evaluated as follows: 131–165 (very high motor ability, VH-MA), 121–130 (high motor ability, H-MA), 111–120 (over average motor capacity, OA-MA), 90–110 (average motor skill, A-MA) 80–89 (lower than average motor skill, UA-MA), 70–79 (low motor capacity, L-MA), and 35–69 (very low motor capacity, VL-MA) [15]. 

### 2.4. Statistical Analysis

Data are shown as means and standard deviations. Normal distribution of the data was verified with Shapiro–Wilk test. The difference within each group was analyzed with Kruskal–Wallis. As the examined variables showed significant differences between the groups before the intervention (age, height, and pre-test scores), the analysis of covariance (ANCOVA) with Tukey–Kramer post hoc was applied using the pre-test scores as covariates. The calculation of the effect size was carried out for ANCOVA analysis through eta squared (η^2^) and for Tukey–Kramer post hoc test through Cohen’s d. In particular, η^2^ = 0.01 indicates a small effect; η^2^ = 0.06 indicates a medium effect; η^2^ = 0.14 indicates a large effect [29]. In regard to Cohen’s d, d = 0.2 is considered a “small” effect size, 0.5 represents a “medium” effect size and 0.8 a “large” effect size. In all the analyses, the level of significance was set at *p* < 0.05. All analyses were performed with Jamovi (The jamovi project (2020) version 1.2 Sydney, Australia) [30]. Retrieved from https://www.jamovi.org (accessed on 26 September 2022) [30].

## 3. Results

**Figure 2:**
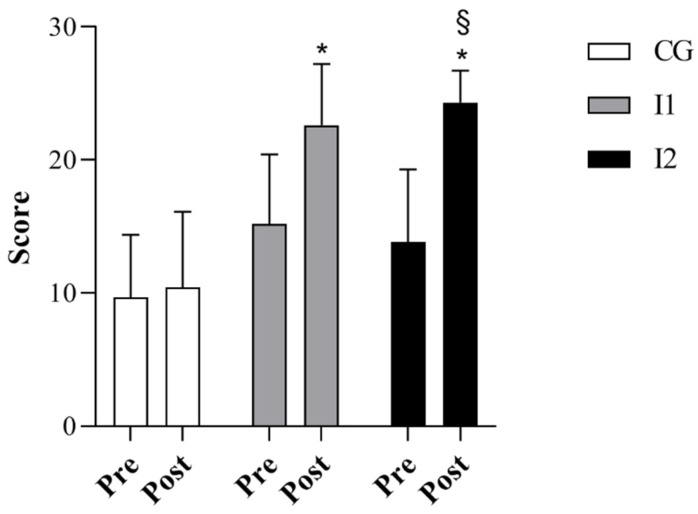
The score of locomotor skills in CG, I1 and I2 groups. * *p* < 0.001 I1 and I2 vs. CG; § *p* < 0.05 I2 vs. I1.

**Figure 3:**
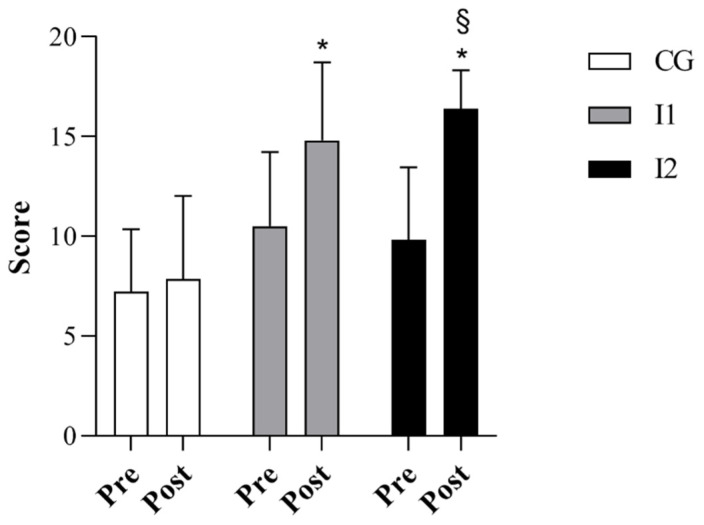
The score of object control skills in CG, I1 and I2 group. * *p* < 0.001 I1 and I2 vs. CG; § *p* < 0.05 I2 vs. I1.

**Figure 4:**
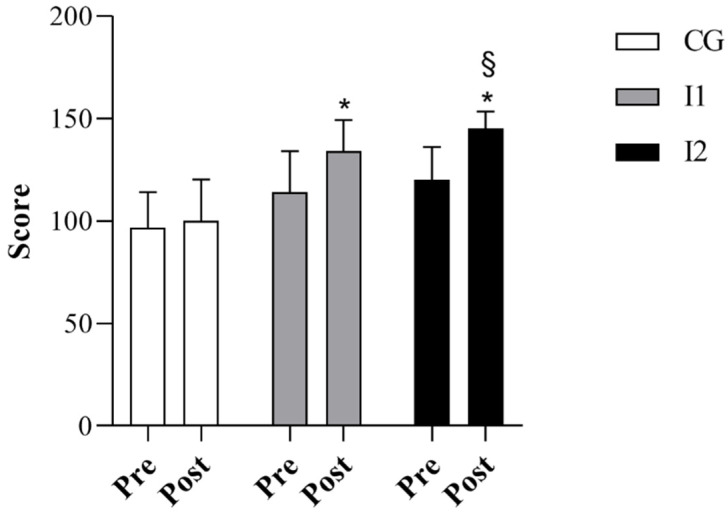
The score of gross motor development quotient (GMDQ) in CG, I1 and I2; * *p*< 0.001 I1 and I2 vs. CG; § *p* < 0.05 I2 vs. I1.

Before the intervention, the CG, I1 and I2 groups did not significantly differ in relation to body weight and BMI. However, age and height were significantly higher in the I1 compared to the I2 group (*p* < 0.05, Table 1), while they did not differ between the CG and both intervention groups. CG did not also show any significant change between pre- and post-measures in locomotion and object control skills and the GMDQ (Figure 2, Figure 3 and Figure 4). Differently, the I1 and I2 groups exhibited significant increases in all three variables examined between pre- and post-intervention (*p* < 0.05, Figure 2, Figure 3 and Figure 4). Comparing the three groups, we found that locomotor skills, object control abilities, and the GMDQ of the I1 and I2 groups was significantly higher than the CG (*p* < 0.001, Figure 2, Figure 3 and Figure 4). In addition, the I2 group displayed a significant increase compared with the I1 children (*p* < 0.05). A large effect of PEP was found for the examined variables as shown by the eta square values, while Cohen’s d indicated a medium effect between the I1 and I2 groups and a large effect between the CG and the intervention groups (see Table 2). In the analysis of the covariate, we did not detect any significant variation between the groups using age or height as covariates.

## 4. Discussion

In this study, we examined the effect that the number of hours engaged in a structured PEP, led by physical education teachers, has on the development of gross motor skills in preschool children. In our previous study [28], the same PEP was performed for a total of 2 h/week, while in this investigation for 4 h/week by the I1 group and for 10 h/week by the I2 group. Our previous results detected a higher percentage in the GMDQ by 10% in the intervention group than the control group, while here we found a better performance by 34% in the I1 group and by 45% in the I2 group compared with the CG. Therefore, this means that more time engaged in a structured physical activity seems to be related with higher levels of motor skills. These findings were confirmed by an increasing number of studies that indicated that greater amounts of PA are associated with better health conditions in preschooler age [3,31]. Recent studies have also shown that in order to prevent obesity and overweight at the preschool age, at least 60 min per day of physical activity are recommended for children between the ages of 3 and 5 years [32,33,34]. However, low and moderate intensity PA was not constantly associated with any health indicators, while moderate to vigorous intensity, vigorous intensity and total physical activity were positively related to more health indicators [2,3,11]. There is a negative relationship between moderate to vigorous physical activity (MVPA) and sedentary behavior, indicating that preschoolers who have higher levels of MVPA have lower levels of sedentary behavior [19]. Several studies have objectively measured the amount of PA through the use of accelerometers, identifying a positive relationship between quantity, development of fundamental movement skills [35,36,37], school performance [35], and quality of sleep [38], showing a negative relationship with the time spent in front of screens [38]. 

For toddlers and preschoolers, the most favorable frequency and duration of physical activity were unclear, but, a greater amount of physical activity appeared to be better for health. However, there is little evidence on the number of hours of school-based physical education that is associated with a higher level of gross motor skills. The goal of physical education in preschool settings is to ensure that children develop the appropriate knowledge, fundamental movement skills, and attitude needed to acquire a healthy lifestyle at an early age, thereby providing them with healthy practices that can later be refined and carried through to adulthood. Furthermore, physical education programs can be educational strategies to improve cognitive learning. Physically active lessons could include teaching content such as math, language, art, and social science, achieving positive effects on physical activity level and learning, results that simple play does not achieve [39].

An increasing number of studies have investigated that structured physical activity programs improve FMS more than do free play activities [18]. There were also significant improvements in preschoolers’ FMS following interventions of physical education led by specialized teachers, although weekly duration and frequency are still unclear to date. In our study, PEP was conducted by a physical education teacher and its features were particularly described, such as the objectives, methodology, type, and quantity of activities. This planning allows to standardize the protocol, which becomes more reproducible. A PEP for a preschool environment is not composed of sets and repetitions of physical exercises but includes games aimed at a specific goal in which simple motor skills become more and more complex. For this reason, we decided to evaluate the amount of PEP by assessing the time dedicated at the development of each examined motor skill. Consequently, we did not use electronic devices (accelerometers and pedometer) to measure the intensity and volume of physical activity as we were more interested in evaluating the quality of the movement. 

A limitation of this study is the presence of a significant difference in age and height between the I1 and I2 groups, which was caused by the dropout due to illness of the children during the intervention. However, in the analysis of the covariate, we did not detect any significant variation between these groups using age or height as covariates. These two variables may not influence the results because the tests administered assess the quality of movement and not the level of physical performance. In addition, despite the fact that the I2 group was the children’ group with the smallest age, it showed the highest level of motor skill performance compared to the others. This phenomenon can be explained by advances and delays in motor and body development of more or less two years in this range of age [40]. Therefore, these results suggest that 10 h/week of PEP are the most effective for the development of motor skills compared with 2 [28] or 4 h/week for schoolers aged 3–5 years old. These findings are also confirmed by the medium-large effect size of PEP on gross motor skills. These results are useful to clarify the dose–response relationship between a structured PEP and the development in motor skills in preschool settings. Furthermore, this standardized PEP can be applied by curricular or physical education teachers for planning effective programs for developing motor skills in early childhood.

Future researches will be to extend the interventions to a larger population and identify the effects of physical education programs on motor skills as well as on lifestyle determinants, physical fitness and cognitive functions.

## Figures and Tables

**Table 1:** Anthropometric characteristics of preschool children.

Group	Age (Years)	Height (cm)	Weight (Kg)	BMI (Kg/m^2^)
CG (*n* = 28)	4.29 ± 0.65	106 ± 6.84	19.5 ± 5.44	17.0 ± 3.14
I1 (*n* = 78)	4.42 ± 0.66	108 ± 7.35	19.8 ± 4.34	16.9 ± 2.64
I2 (*n* = 39)	3.92 ± 0.83 *	104 ± 6.88 *	17.6 ± 3.59	16.2 ± 2.11

BMI, body mass index. * *p* < 0.05 I1 vs. I2.

**Table 2:** Measures of effect size in ANCOVA analysis and Tukey–Kramer post hoc test.

	Locomotor Skills			Object Control Skills			GMDQ		
**ANCOVA**	**F**	***p***	**η^2^**	**F**	***p***	**η^2^**	**F**	***p***	**η^2^**
	90.0	<0.001	0.561	42.6	<0.001	0.377	60.0	<0.001	0.460
	**CG vs. I1**	**CG vs. I2**	**I1 vs.** **I2**	**CG vs. I1**	**CG vs. I2**	**I1 vs.** **I2**	**CG vs.** **I1**	**CG vs.** **I2**	**I1 vs.** **I2**
**ptukey**	<0.001	<0.001	0.005	<0.001	<0.001	0.011	<0.001	<0.001	0.002
**Cohen’s d**	2.695	3.330	0.635	1.733	2.312	0.579	2.181	2.866	0.686

## Data Availability

The data presented in this study are available on request from the corresponding author. The data are not publicly available due to the young age of the participants.

## References

[1] Diaz-Castro J., Garcia-Vega J.E., Ochoa J.J., Puche-Juarez M., Toledano J.M., Moreno-Fernandez J. (2021). Implementation of a Physical Activity Program Protocol in Schoolchildren: Effects on the Endocrine Adipose Tissue and Cognitive Functions. Front. Nutr..

[2] Chaput J.P., Willumsen J., Bull F., Chou R., Ekelund U., Firth J., Jago R., Ortega F.B., Katzmarzyk P.T. (2020). 2020 WHO guidelines on physical activity and sedentary behaviour for children and adolescents aged 5–17 years: Summary of the evidence. Int. J. Behav. Nutr. Phys. Act..

[3] Tremblay M.S., Chaput J.P., Adamo K.B., Aubert S., Barnes J.D., Choquette L., Duggan M., Faulkner G., Goldfield G.S., Gray C.E. (2017). Canadian 24-Hour Movement Guidelines for the Early Years (0–4 years): An Integration of Physical Activity, Sedentary Behaviour, and Sleep. BioMed Cent. Public Health.

[4] Brown W.H., Pfeiffer K.A., McIver K.L., Dowda M., Addy C.L., Pate R.R. (2009). Social and environmental factors associated with preschoolers’ nonsedentary physical activity. Child Dev..

[5] Gubbels J.S., Kremers S.P., van Kann D.H., Stafleu A., Candel M.J., Dagnelie P.C., Thijs C., de Vries N.K. (2011). Interaction between physical environment, social environment, and child characteristics in determining physical activity at child care. Health Psychol. Off. J. Div. Health Psychol. Am. Psychol. Assoc..

[6] Dowda M., Pate R.R., Trost S.G., Almeida M.J., Sirard J.R. (2004). Influences of preschool policies and practices on children’s physical activity. J. Community Health.

[7] Konstabel K., Veidebaum T., Verbestel V., Moreno L.A., Bammann K., Tornaritis M., Eiben G., Molnár D., Siani A., Sprengeler O. (2014). Objectively measured physical activity in European children: The IDEFICS study. Int. J. Obes..

[8] https://www.istat.it/it/files//2019/10/Report_Stili_di_vita_minori.pdf.

[9] Navarra G.A., Thomas E., Scardina A., Izadi M., Zangla D., De Dominicis S., Cataldo P., Proia P., Bellafiore M. (2021). Effective Strategies for Promoting Physical Activity through the Use of Digital Media among School-Age Children: A Systematic Review. Sustainability.

[10] Jáuregui A., Argumedo G., Medina C., Bonvecchio-Arenas A., Romero-Martínez M., Okely A.D. (2021). Factors associated with changes in movement behaviors in toddlers and preschoolers during the COVID-19 pandemic: A national cross-sectional study in Mexico. Prev. Med. Rep..

[11] Janssen I., Leblanc A.G. (2010). Systematic review of the health benefits of physical activity and fitness in school-aged children and youth. Int. J. Behav. Nutr. Phys. Act..

[12] Nielsen-Rodríguez A., Romance R., Dobado-Castañeda J.C. (2021). Teaching Methodologies and School Organization in Early Childhood Education and Its Association with Physical Activity. Int. J. Environ. Res. Public Health.

[13] Hardy L.L., King L., Kelly B., Farrell L., Howlett S. (2010). Munch and Move: Evaluation of a preschool healthy eating and movement skill program. Int. J. Behav. Nutr. Phys. Act..

[14] Battaglia G., Giustino V., Tabacchi G., Lanza M., Schena F., Biino V., Giuriato M., Gallotta M.C., Guidetti L., Baldari C. (2021). Interrelationship Between Age, Gender, and Weight Status on Motor Coordination in Italian Children and Early Adolescents Aged 6-13 Years Old. Front. Pediatr..

[15] Ulrich D.A. (1992). Test TGM.

[16] Gallahue D.L., Donnelly F.C. (2007). Developmental Physical Education for All Children.

[17] Lloyd M., Saunders T.J., Bremer E., Tremblay M.S. (2014). Long-term importance of fundamental motor skills: A 20-year follow-up study. Adapt. Phys. Act. Q..

[18] Logan S.W., Robinson L.E., Wilson A.E., Lucas W.A. (2012). Getting the fundamentals of movement: A meta-analysis of the effectiveness of motor skill interventions in children. Child Care Health Dev..

[19] Engel A.C., Broderick C.R., van Doorn N., Hardy L.L., Parmenter B.J. (2018). Exploring the Relationship Between Fundamental Motor Skill Interventions and Physical Activity Levels in Children: A Systematic Review and Meta-analysis. Sport. Med..

[20] Diamond A. (2015). Effects of Physical Exercise on Executive Functions: Going beyond Simply Moving to Moving with Thought. Ann. Sport. Med. Res..

[21] Alesi M., Bianco A., Luppina G., Palma A., Pepi A. (2016). Improving Children’s Coordinative Skills and Executive Functions: The Effects of a Football Exercise Program. Percept. Mot. Ski..

[22] Rhemtulla M., Tucker-Drob E.M. (2012). Gene-by-socioeconomic status interaction on school readiness. Behav. Genet..

[23] Oberer N., Gashaj V., Roebers C.M. (2017). Motor skills in kindergarten: Internal structure, cognitive correlates and relationships to background variables. Hum. Mov. Sci..

[24] Goodway J.D., Ozmun J.C., Gallahue D.L. (2019). Understanding Motor Development: Infants, Children, Adolescents, Adults.

[25] Petrigna L., Thomas E., Scardina A., Rizzo F., Brusa J., Camarazza G., Galassi C., Palma A., Bellafiore M. (2022). Methodological Considerations for Movement Education Interventions in Natural Environments for Primary School Children: A Scoping Review. Int. J. Environ. Res. Public Health.

[26] Szeszulski J., Lanza K., Dooley E.E., Johnson A.M., Knell G., Walker T.J., Craig D.W., Robertson M.C., Salvo D., Kohl H.W. (2021). Y-PATHS: A Conceptual Framework for Classifying the Timing, How, and Setting of Youth Physical Activity. J. Phys. Act. Health.

[27] https://www.miur.gov.it/documents/20182/51310/DM+254_2012.pdf/1f967360-0ca6-48fb-95e9-c15d49f18831?version=1.0&t=1480418494262.

[28] Battaglia G., Alesi M., Tabacchi G., Palma A., Bellafiore M. (2018). The Development of Motor and Pre-literacy Skills by a Physical Education Program in Preschool Children: A Non-randomized Pilot Trial. Front. Psychol..

[29] Cohen J. (2013). Statistical Power Analysis for the Behavioral Sciences.

[30] Love J., Dropmann D., Selker R., Gallucci M., Jentschke S., Balci S., Seol H., Agosti M. (2020). The jamovi Project. jamovi (Version 1.2) [Computer Software]. https://www.jamovi.org.

[31] Barnett L.M., Lai S.K., Veldman S.L.C., Hardy L.L., Cliff D.P., Morgan P.J., Zask A., Lubans D.R., Shultz S.P., Ridgers N.D. (2016). Correlates of Gross Motor Competence in Children and Adolescents: A Systematic Review and Meta-Analysis. Sport. Med..

[32] Weihrauch-Blüher S., Kromeyer-Hauschild K., Graf C., Widhalm K., Korsten-Reck U., Jödicke B., Markert J., Müller M.J., Moss A., Wabitsch M. (2018). Current Guidelines for Obesity Prevention in Childhood and Adolescence. Obes. Facts.

[33] Reilly J.J., Kelly L., Montgomery C., Williamson A., Fisher A., McColl J.H., Lo Conte R., Paton J.Y., Grant S. (2006). Physical activity to prevent obesity in young children: Cluster randomised controlled trial. BMJ (Clin. Res. Ed.).

[34] Foster C., Moore J.B., Singletary C.R., Skelton J.A. (2018). Physical activity and family-based obesity treatment: A review of expert recommendations on physical activity in youth. Clin. Obes..

[35] Jones D., Innerd A., Giles E.L., Azevedo L.B. (2021). The Association between Physical Activity, Motor Skills and School Readiness in 4-5-Year-Old Children in the Northeast of England. Int. J. Environ. Res. Public Health.

[36] Jones D., Innerd A., Giles E.L., Azevedo L.B. (2020). Association between fundamental motor skills and physical activity in the early years: A systematic review and meta-analysis. J. Sport Health Sci..

[37] Kracht C.L., Webster E.K., Staiano A.E. (2020). Relationship between the 24-Hour Movement Guidelines and fundamental motor skills in preschoolers. J. Sci. Med. Sport.

[38] Webster E.K., Martin C.K., Staiano A.E. (2019). Fundamental motor skills, screen-time, and physical activity in preschoolers. J. Sport Health Sci..

[39] Petrigna L., Roggio F., Trovato B., Zanghì M., Musumeci G. (2022). Are Physically Active Breaks in School-Aged Children Performed Outdoors? A Systematic Review. Sustainability.

[40] Malina R.M. (2004). Motor Development during Infancy and Early Childhood: Overview and Suggested Directions for Research. Int. J. Sport Health Sci..

